# Effect of Micro-Nano Bubble Water and Silica Fume on Properties of C60 Concrete

**DOI:** 10.3390/ma16134684

**Published:** 2023-06-29

**Authors:** Shuang He, Tingshu He, Zhenmin Wan, Qing Zhao

**Affiliations:** Faculty of Materials Science and Engineering, Xi’an University of Architecture and Technology, Xi’an 710064, China; zhenminwan@xauat.edu.cn (Z.W.); zhaoqing@xauat.edu.cn (Q.Z.)

**Keywords:** micro-nano bubble water, silica fume, working performance, compressive strength, durability

## Abstract

Micro-nano bubble water (WNBW) in concrete is relatively uncommon due to its newness as a technology. This paper presents the preparation of C60 concrete with 35% fly ash (FA) through WNBW and varying amounts of silica fume (0%, 4%, 7%, and 10% SF). The study examines the impact of WNBW and SF on the working performance, compressive strength, and durability of concrete. The findings indicate that applying WNBW and SF independently or jointly deteriorates the working performance of fresh concrete. However, compared to regular mixing water, WNBW reduces the concrete passing time through the V-funnel, decreasing by 40%, 39.1%, 42.9%, and 50.5% for the four varying SF contents. Furthermore, using WNBW, SF, or both resulted in the increased compressive strength of concrete at 7 days and 28 days, with 7% SF content yielding a 12.2% and 6.6% increase, respectively. Using a combination of WNBW and SF has been shown to decrease the impermeability of concrete effectively. The addition of 4% SF results in the lowest electric flux when using regular mixing water, with a discernible decrease of 30.1% compared to the control group. Conversely, using WNBW as mixing water yields a decrease in electric flux at each SF content, with the maximum decrease being 39.7%. Furthermore, both the single and combined use of these materials can contribute to the reduction in the carbonation resistance of the concrete. C60 concrete mixed with 7% SF and 100% WNBW boasts enhanced frost resistance, as indicated by the mass loss and dynamic elastic modulus loss being the least following freeze–thaw under the same SF content. According to the findings of the tests, there is evidence that the incorporation of 7% SF and 100% WNBW into C60 concrete results in lowered viscosity, a highly advantageous attribute for actual construction. Additionally, this mixture displays impressive compressive strength and durability properties. These results provide technical support regarding the integration of WNBW and SF in C60 concrete.

## 1. Introduction

To reduce global carbon emission pollution, all industries are facing the problem of energy conservation and emission reduction. Studies have shown that the cement industry accounts for 5% of global CO_2_ emissions [1,2]. With national infrastructure development, the demand for cement will continue to increase. Reducing the number of cementitious materials has become the future direction of the development of the commercial concrete industry. More and more researchers have begun to study the methods of supplementing cementitious materials to improve the performance of concrete.

Silica fume (SF) is a finely-grained material that is a by-product of ferrosilicon alloy or silicon metal production in the metallurgical industry. With its amorphous SiO_2_ content reaching as high as 90%, the material has a substantial specific surface area, with predominantly nano-scale particles, and boasts exceptionally high pozzolanic activity [3,4]. Its primary applications include the preparation of high-strength [5,6], ultra-high-strength [7,8], and high-viscosity concrete, such as underwater non-dispersed concrete, with a recommended content of 5% to 10% of the total cementitious materials involved. Research has proven that the addition of SF is beneficial in improving the compressive strength and durability of concrete, enhancing the concrete’s density, reducing the pore count and size, and improving the transition zone interface. Zhang Heng-yuan et al. [9] conducted a study to analyse the impact of SF content (0%, 3%, 6%, 9%, and 13%) on high-performance concrete with a low water–cement ratio (w/c = 0.28). The results indicate an initial increase followed by a decrease in the slump, expansion rate, compressive strength, and flexural strength of the concrete with an increase in SF content. Optimal working performance is observed at 3% SF content, while the most significant strength increase is seen in the range of 3% to 6% SF content. When the SF content exceeds 9%, there is a significant reduction in slump and only a slight decrease in strength. However, the small particle size and high surface energy of SF cause easy aggregation [10,11,12], weakening performance. Dongyeop Han et al. [13] conducted a study to compare the advantages and disadvantages of pre-mixing [13] and traditional stirring methods by pre-mixing SF powder. The results show that pre-mixing improves the dispersion of powder, water, and water-reducing admixture during the cement base formation process. Additionally, SEM analysis indicates that pre-mixing SF dispersion is more uniform and agglomeration is reduced compared to traditional mixing methods.

Micro-nano bubble water (WNBW) has generated considerable interest as a promising concrete-mixing water. It features a high concentration of micro-nano bubbles with diameters ranging from tens of microns to hundreds of nanometres [14]. These bubbles possess a large specific surface area, slow rise in water, negatively charged surface [15], high ζ potential, high mass transfer efficiency, and high stability [16,17] and activity. By forming a stable electric double-layer structure in water [18], micro-nano bubbles can adsorb SF particles and impart the same charge to their surface, thus mitigating SF agglomeration [19]. Further, micro-nano bubbles can lower the surface tension of water, enhancing its wettability on solid surfaces and facilitating contact and dispersal with SF particles. Incorporating WNBW in cementitious material ensures the homogeneous mixing of the SF particles with other constituents. Additionally, WNBW promotes cement hydration and augments the early strength of concrete, supplementing the reduced early strength performance of concrete variants with lower cement content [20,21,22,23]. Zhenmin Wan et al. [24] use WNBW in place of regular water and combined it with an alkali-free liquid accelerator. The results indicated that the addition of WNBW significantly decreased the initial and final setting of cement paste while also improving the 1 d and 7 d compressive strength of cement mortar without any subsequent shrinkage in strength. Further analysis using various techniques such as SEM, XRD, and hydration temperature testing revealed that the use of WNBW as mixing water for cement mortar led to an accelerated hydration process for cement-based materials. The WNBW facilitated a faster reaction of C_3_S and C_3_A, leading to a higher peak value of early hydration temperature and the improved compactness and uniformity of the hardened cement stone’s structure. The researchers then prepared C25 grade shotcrete using micro-nano bubble water as mixing concrete water and tested its frost resistance, finding that the WNBW reduced the concrete pore size, increased its density, and strengthened the connection between the cement paste and aggregate, thus significantly enhancing the frost resistance of the shotcrete.

Previous scholarly research indicates that the impact of utilizing WNBW to enhance the operational proficiency of SF concrete has not been explored. Additionally, the issue of diminishing viscosity in high-strength concrete has yet to be examined. Therefore, there is a need for further investigation of the influence of WNBW and SF on the working performance, compressive strength, and durability of high fly ash (FA) C60 concrete. This research uses 35%FA to replace cement and evaluates the impact of altering the mixing water (using ordinary water and bubble water) and SF content on the performance of C60 concrete with high FA. Through multiple tests, including slump, expansion, compressive strength, electric flux, carbonation, and rapid freeze–thaw, this study investigates the influence and reasoning behind the effects of WNBW and SF on concrete performance. The findings of this research provide valuable guidance on the combined utilisation of WNBW and SF.

## 2. Materials and Methods

### 2.1. Materials and Mixing Water

#### 2.1.1. Materials

The materials used in this study, namely, P·O 42.5 grade cement, II grade FA, compacted SF, and S95 grade slag, were procured from China Construction Western Construction North Co., Ltd. These materials were compliant with national standards [25] and underwent X-ray fluorescence analysis to ensure their quality. The chemical composition of the cement, FA, SF, and ground granulated blast furnace slag (GGBS) was determined and is presented in Table 1.

In this experimental study, the fine aggregate chosen was river sand, while the coarse aggregate was stone, which were both procured from Shaanxi. The physical attributes of these materials are meticulously documented in Table 2 and Table 3.

#### 2.1.2. WNBW Preparation

This study utilised pure tap water without impurities with a pH value of 7.1. To produce WNWB, ordinary tap water was used. Throughout the production process, the intake of air was adjusted to sustain a consistent flow at 1 L per minute, while the instrument pressure was regulated to approximately 0.5 ± 0.1 MPa [26]. Typically, the preparation process takes about 5 min. The freshly produced WNBW appears milky white due to the dispersion of micro-nano bubbles in the water, resulting in an emulsion formation. These tiny micro-nano bubbles measure between a few nanometres and microns in diameter, typically not visible to the naked eye, but they scatter light and create a milky white tone in the water. Eventually, the micro-sized bubbles disappeared after 2 to 3 min, leaving the WNBW in the same transparent and clear state as the ordinary water. To ensure test consistency, the produced WNBW water was allowed to stand for half an hour before mixing with concrete. This particular apparatus is a specialised tool designed for the production of micro-nano bubble water. The accompanying parameter table indicates that the smallest diameter achievable for the micro-nano bubbles produced by the instrument is between 10 and 100 nanometres [27].

The process for producing micro-nano bubble water involves the mixing and high-speed rotation of gas and liquid under pressure. This generates a negative pressure axis within the generator. Upon ejection from the nozzle at a specific pressure, the high-speed and strong shear, combined with high-frequency pressure change at the gas–liquid contact interface, creates artificial extreme conditions. These conditions lead to the generation of a significant number of micro-nano bubbles. A description of the mechanism has been added to the revision.

### 2.2. Mix Proportion

The present study focuses on mixing C60 concrete with varying proportions of SF (0%, 4%, 7%, 10%) and FA (35%). The mixing water employed during the process includes ordinary tap water and WNBW. The mix design incorporates a water-binder ratio of 0.31 for C60 concrete, with a pumping agent proportion of 2.1% of the total cementitious material. Furthermore, the sand ratio is 43%, and the design slump is 200 ± 20 mm. The detailed breakdown of the mix design is provided in Table 4.

### 2.3. Testing Method

#### 2.3.1. Concrete Workability Test

The workability test refers to the specification GB/T50080-2016 [28] to determine the working performance of the concrete mixture and carry out a concrete slump test and expansion time loss test.

#### 2.3.2. Compressive Strength of Concrete

The experiment involved the evaluation of the cube compressive strength of concrete for 7 and 28 days, referencing the standard GB/T50081-2019 [29]. The dimensions of the specimen mould measured 100 mm × 100 mm × 100 mm. Following removal from the mould, the specimens were transferred to a humidity-controlled room with a minimum humidity of 95% and maintained at 20 ± 2 °C for curing.

#### 2.3.3. Concrete Impermeability Test

The GB/T50082-2009 [30] standard is utilised for determining the long-term performance and durability of ordinary concrete through the application of the electric flux method. The specimen is formed to a diameter of 100 mm and height of 50 mm, and the test commences at 28 days old. Following drying, the resin is used to seal the side of the cylinder and all holes are filled. Vacuum water retention with deionised water is undertaken and placement in the test tank is performed with a positive electrode solution of 3% NaCl and a negative electrode solution of 0.3 mol/L NaOH concentration. The instrument then automatedly performs the testing and recording of data. The evaluation of the electric flux value is the technique utilised to determine the impermeability of concrete [31,32].

#### 2.3.4. Concrete Carbonation Resistance Test

This experiment follows the specifications outlined in the GB/T50082-2009. The specimen, measuring dimensions of 100 mm × 100 mm × 100 mm, was extracted two days prior to the evaluation. They were then subjected to a temperature of 60 °C for 48 h, leaving two corresponding sides unsealed. The remaining surfaces were sealed with heated paraffin wax, cooled, and placed in a concrete carbonisation test chamber where the CO_2_ concentration was maintained at 20 ± 1%, humidity at 90 ± 5%, and temperature at 20 ± 2 °C. At 3 d, 7 d, 14 d, and 28 d, the specimens were removed from the chamber and subjected to splitting tests using a pressure-testing machine. The carbonisation depth was determined through the use of 1% phenolphthalein alcohol solution.

#### 2.3.5. Concrete Frost-Resistance Test

This study used the established GB/T50082-2009, employing the rapid freezing technique to conduct freeze–thaw trials on concrete specimens measuring 100 mm × 100 mm × 400 mm. The concrete samples were extracted after 24 days of curing, submerged in water at 20 ± 2 °C, and subjected to freeze–thaw trials at 28 days of curing. The investigation monitored the mass loss and relative dynamic elastic modulus loss at 25 freeze–thaw cycle intervals. A total of 200 freeze–thaw cycles were included.

#### 2.3.6. Analysis Test of Freeze–Thaw Concrete Pore Structure

Preparing the pore analysis sample involved cutting a 100 mm × 100 mm × 10 mm sheet from the concrete test block using a mechanical process, and then polishing with sandpaper. To ensure the complete impregnation of all concrete pores with black ink, the concrete cutting interface was coated evenly with this ink. Subsequently, pure white nano barium sulphate (BaSO_4_) was applied uniformly to the concrete’s cutting section and any excess material was retrieved. For the porosity analysis sample testing, the concrete test block was positioned within the sample chamber of the NELD-BS610 hardened concrete pore analyser. The microscope magnification was adjusted manually to display a clear image on the computer. A scanning area of 50 mm × 50 mm was determined and tested. In a short duration ranging from 3 to 5 min, the instrument captured an image automatically, and the NELD-BS610 bubble analysis software (TYC-457) issued an electronic conclusion book that included vital parameters such as bubble spacing coefficient, pore specific surface area, gas content, cementitious material, porosity, average chord length of bubbles, the average radius of bubbles, and number of bubbles.

#### 2.3.7. V-Funnel through Time

In order to perform the V-shaped funnel test, it was necessary to clean the funnel meticulously and place it onto a level plane. Subsequently, the internal surface of the funnel was moistened with a damp cloth, and a receptacle was arranged underneath it to gather the cement mixture. The procedure involved pouring the mixture onto the funnel, followed by allowing it to rest for a duration of 10 ± 2 s. Subsequently, the bottom cover of the funnel was eliminated, and the duration for which the mixture took to empty was recorded with a precision of 0.1 s. It is important to mention that the accurate instant when light is visibly emanating from the upper extremity of the funnel is deemed to be the instance of concrete drainage.

## 3. Results and Discussion

### 3.1. Working Performance

**Figure 1:**
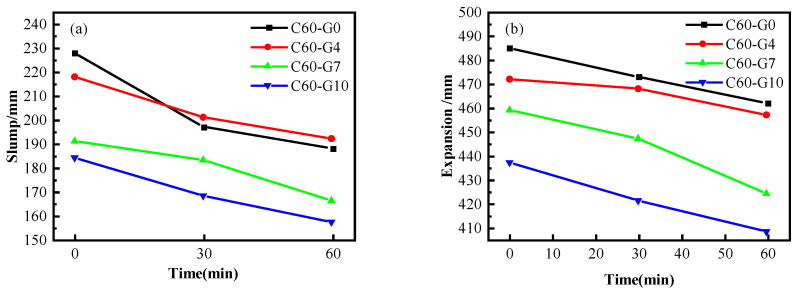
Influence of SF on working performance of C60 concrete: (**a**) slump; (**b**) slump flow.

Figure 1a,b display the impact of SF on the slump, expansion, and time loss for C60 concrete containing 35% FA. As observed, when using ordinary water as the mixing water, the slump and expansion of C60 concrete decrease with increasing SF content. The initial slump of C60-G4, C60-G7, and C60-G10 decreased by 44 mm, 34 mm, and 7 mm, respectively, in comparison to C60-G0. After 30 min, the slump of C60-G4, C60-G7, and C60-G10 decreased by 29 mm, 33 mm, and 15 mm, respectively, compared to C30-G0. Similarly, after 60 min, the slump of C60-G4, C60-G7, and C60-G10 decreased by 31 mm, 35 mm, and 9 mm, respectively, in comparison to C30-G0. The effect of SF on the flow performance of C60 concrete is negative, as the pattern of expansion follows the trend of slump.

**Figure 2:**
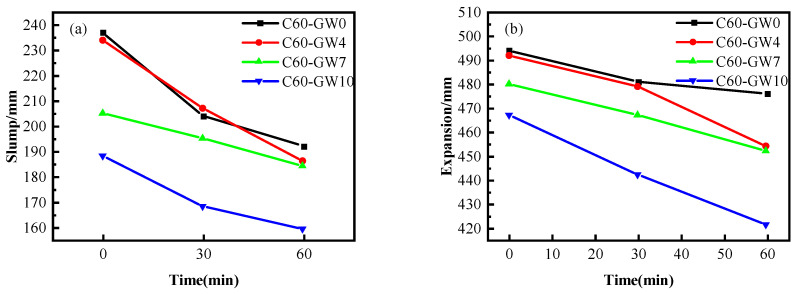
Effect of the composite use on the workability of C60 concrete: (**a**) slump; (**b**) slump flow.

The results showcased in Figure 2a,b depict the impact of WNBW and SF on the slump, expansion, and time loss of C60 concrete containing 35% FA. The figure substantiates that when WNBW and SF were used simultaneously, the slump and expansion of C60 concrete decreased progressively with the increase in SF proportion. Additionally, it is noticeable that WNBW failed to enhance the flow performance of the concrete. The performance of the concrete was suboptimal when the SF content was at 10%, with an initial slump and expansion of 184 mm and 437 mm, and 1 h slump and expansion of 157 mm and 408 mm, respectively. Notably, WNBW had minimal impact on the working performance of C60 concrete compared to ordinary water-mixed concrete, accompanied by lower 1 h loss.

In conjunction with Figure 1 and Figure 2, it is evident that the inclusion of both WNBW and SF adversely impacts the fluidity of concrete. One possible explanation is that SF has a small particle size and a large specific surface area, which allows for the adsorption of significant amounts of water molecules during the mixing process [33]. Another possibility is that WNBW contains a substantial number of hydroxyl radicals and has a high pH, contributing to a higher ζ point and an increased probability of collision with cement particles [15]. These factors can expedite the early hydration of cement, resulting in the reduced workability of the concrete. Consistent with the findings of Hassani et al. [21], WNBW has been shown to shorten the initial and final setting times and decrease the flow performance of the cement mortar.

### 3.2. V-Funnel through Time

During the experiment, it was observed that C60 concrete with an FA content of 35% exhibited high viscosity and complex mixing, which worsened with an increase in SF content. Table 5 demonstrates that as the SF content increased in the concrete mixed with ordinary water, the passage time through the V-funnel gradually increased. At 10% SF content, the passage time increased by 38.7 s. In contrast, when using WNBW for the mixing water, the passage time remained approximately 70 s. Furthermore, compared to the ordinary water mix, the passage time with WNBW reduced by 40%, 39.1%, 42.9%, and 50.5% for the same SF content levels. These results indicate that WNBW played a crucial role in reducing the passage time of the concrete V-funnel, enhancing the pumping performance of the concrete [34] and minimising the phenomenon of concrete bottom grabbing. Numerous micro-nano bubbles in WNBW evenly distributed in the fresh concrete acted as rolling bearing beads, consequently reducing the friction of the concrete during flow [20].

### 3.3. Compressive Strength

**Figure 3:**
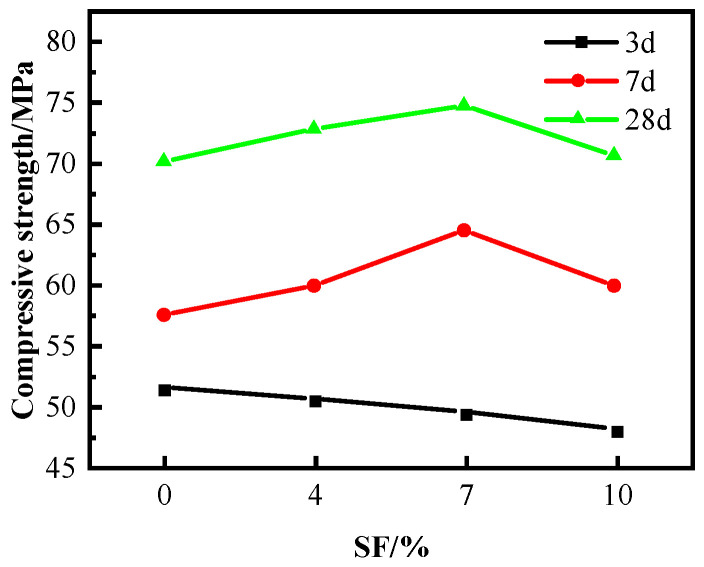
Effect of SF on compressive strength of C60.

As shown in Figure 3, the findings reveal that the three-day compressive strength of the concrete declines in proportion to the increase in SF content. However, when the SF content is at 7%, the concrete’s 7-day and 28-day compressive strength reaches its peak, exhibiting improvements of 12.2% and 6.6%, respectively, compared to the control group that lacked SF.

**Figure 4:**
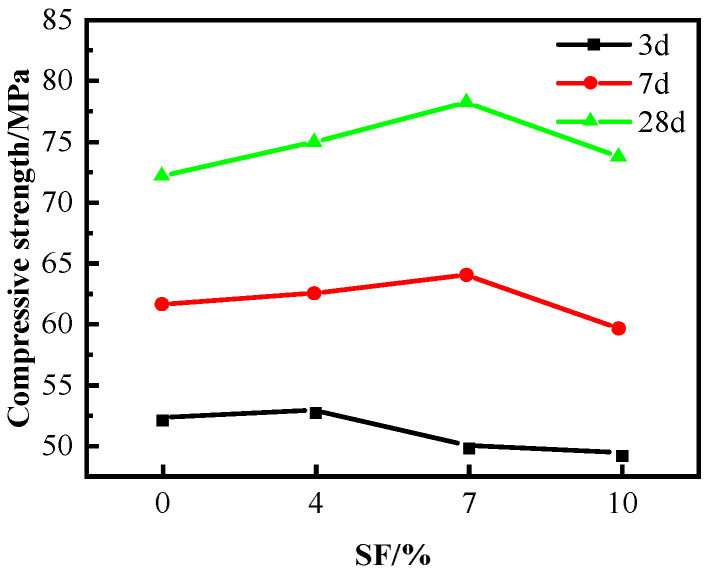
Effect of composite use on the compressive strength of C60.

The results depicted in Figure 4 demonstrate the impact of SF on the compressive strength of C60 concrete wherein WNBW acts as the mixing water. The findings reveal that the concrete’s 7 d and 28 d compressive strength variation mirrors that of the conventional water-mixed concrete, and it depreciates with an upsurge in SF proportion. The concrete’s 7 d and 28 d compressive strength is recorded as 63.9 MPa and 78.2 MPa, respectively, at an SF content of 7%. The possible explanation for the decline in 3 d concrete strength is that the increase in SF and 35% FA content reduces the amount of cement [8], thereby generating less C-S-H during the initial hydration stage, resulting in a loose concrete structure and reduced strength.

### 3.4. Impermeability of Concrete

Table 6 presents an analysis of the impact of WNBW and SF on the electric flux of C60 concrete. It can be found that, in the case of regular water mixing, the electric flux of C60 concrete initially decreased and then increased upon adding SF from 0% to 10%. The electric flux value significantly decreases with an SF content of 4%, resulting in a recorded minimum value of 363.56 C. This represents a substantial decline of 48.9% compared to the control group, composed of concrete without SF, and experienced a 347.56 C decrease in the electric flux value. As the SF content increased, the electric flux value gradually increased. Concrete containing 7% and 10% SF demonstrated electric flux values of 403.08C and 422.64C, respectively, which were lower than the control group.

When using WNBW as the mixing water, the electric flux value of C60 concrete initially decreases and subsequently increases as the SF content increases. Upon an SF content of 4%, the concrete’s resulting electric flux value is observed at 321.32 C. A comparative analysis with the control group indicates a decrease of 49.9%, totalling a decrement of 320.44 C. Further research concerning the impact of SF content on electric flux values highlights that an increased SF content of 7% and 10% leads to respective increases in electric flux values at 405.68 C and 489.72 C, which do not diverge significantly from the control group.

Furthermore, it has been observed that the electronic flux rating in concrete containing the same level of SF is generally lower [35,36] when compared to C60 concrete produced using regular water, with the exception of SF content at 10%. This phenomenon is attributed to the stable solid–liquid interface generated by the introduction of micro-nano bubbles in the concrete, leading to better mixing of SF particles with other elements in the concrete and a uniform distribution throughout the structure [37]. Furthermore, WNBW, owing to its high dispersion and permeability properties, facilitates the dispersion and dissolution of various admixtures in concrete, ensuring the even distribution of constituents [38]. These findings highlight the benefits of using WNBW and SF, both separately and in combination, in enhancing the compaction and chloride ion erosion resistance of C60 concrete.

### 3.5. Carbonation Resistance of Concrete

**Figure 5:**
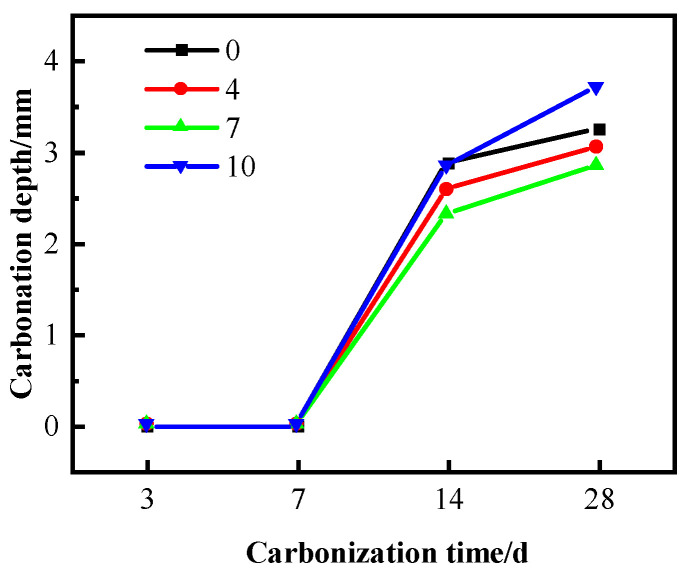
Carbonation depth of tap-water-mixed concrete.

Figure 5 shows the effect of SF on the carbonation resistance of C60 concrete mixed with tap water. The carbonation depth of concrete samples containing 0%, 4%, 7%, and 10% SF mixed with ordinary water, was determined through rapid carbonation for 3 and 7 days. The results indicated that concrete with a low water–binder ratio possessed a dense internal structure, lower calcium hydroxide content, and a less conspicuous carbonation effect. However, after 14 and 28 days of rapid carbonisation, an increase in SF content led first to an increase and then a decrease in the carbonation depth [39,40]. Specifically, the carbonation depth of concrete containing 7% SF was reduced by 10.1% and 12.3% at 14 and 28 days, respectively.

**Figure 6:**
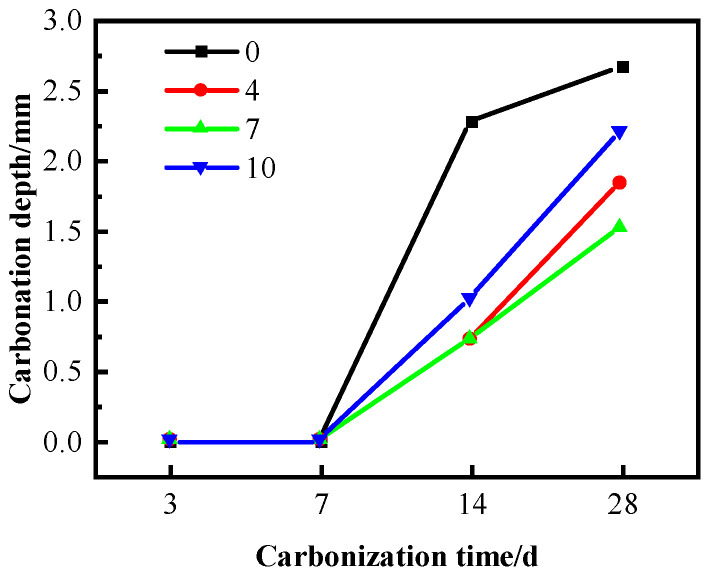
Carbonation depth of WNBW-mixed concrete.

Figure 6 shows the effect of SF on the carbonation resistance of C60 concrete mixed with WNBW. The concrete samples mixed with WNBW showed a carbonation depth of 0 after 3 and 7 days of rapid carbonation. Subsequently, the carbonation depth increased after 14 and 28 days, with a significant decrease compared to those mixed with ordinary water. This can be attributed to the uniformity of surface slurry and reduced agglomeration deposition of different powders resulting from incorporating WNBW during the curing process. Additionally, the carbonation depth of the sample surpassed the surface layer depth after 14 and 28 days of rapid carbonation. The impact of SF content on carbonation depth also changed significantly, with a minimum carbonation depth of 1.48 mm for 28 days observed when the SF content was at 7%.

In C60 concrete, the observations indicate that the carbonation depth initially experiences a decrease, followed by an increase with a corresponding increase in the SF content. A noteworthy finding reveals that SF plays a significant role in reducing the overall carbonation depth of concrete to varying degrees, and coupling with WNBW, SF can supplementarily improve the carbonation resistance of concrete.

### 3.6. Frost Resistance of Concrete

#### 3.6.1. Mass Loss

**Figure 7:**
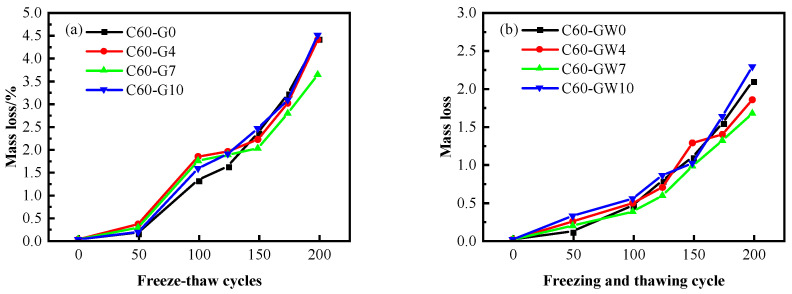
Mass loss of concrete after freeze–thaw: (**a**) ordinary water, (**b**) micro-nano bubble water.

As the frequency of freeze–thaw cycles rises, there is a gradual increase in the mass loss sustained by the concrete specimens. Despite some gradual loss of cement paste from the concrete surface, C60 concrete specimens display relatively minimal mass loss and no aggregate exposure. Concrete specimens with a low water–binder ratio experience tighter bonding between cement stone and aggregate, leading to less damage caused by the freeze–thaw phenomenon. The provided Figure 7 indicates that in the C60 concrete, the mass loss remained stable, roughly at 4%, after 200 freeze–thaw cycles.

In testing the effect of SF content on the frost resistance of C60 concrete, ordinary water was used as the mixing water. The results showed that after 200 freeze–thaw cycles, the mass loss of the concrete initially decreased and then increased with increased SF content. The optimal frost resistance was observed at an SF content of 7%, with a mass loss of 3.64%, 17.5% lower than the blank group. However, at 4% and 10% SF contents, the mass loss was greater than that of C60-G7. Furthermore, the mass loss rate of C60-G10 was slightly higher than that recorded for the blank group.

In cases where WNBW serves as the mixing water for C60 concrete, the mass loss rule follows a pattern similar to conventional water-mixed C60 concrete. At 7% SF content, the concrete experiences a minimum mass loss of 1.67%, while at 10% SF content, the maximum mass loss recorded does not exceed 2.41%.

Upon comparing the mixing water of WNBW and ordinary water concrete, it was determined that the mass loss rate of C60 concrete is lowest at 7% SF content. As for C60 concrete, the mass loss rates under different mixing conditions were observed to be 3.64% and 1.67%, respectively. The utilisation of WNBW as the concrete mixing water was proven to exhibit excellent macroscopic performance. Upon subjecting the concrete to freezing and thawing, the outer surface remained relatively flat, demonstrating minimal damage to the eight corners of the test block, with only some mortar falling off. This suggests that the incorporation of WNBW serves to reduce the mass loss of the outer surface of the concrete after freeze–thaw, and its effect on high-strength concrete is particularly prominent. Such a positive outcome is owed to the excellent dispersion effect and chemical activity of micro-nano bubbles, which promote the uniform distribution of cementitious materials and enhance the concrete strength.

#### 3.6.2. Relative Dynamic Elastic Modulus

**Figure 8:**
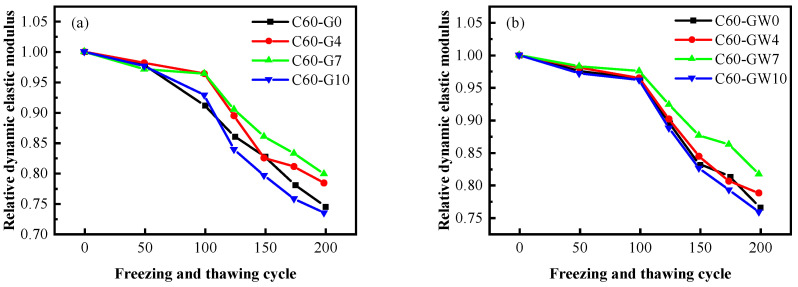
Relative dynamic elastic modulus of concrete after freeze–thaw cycles: (**a**) ordinary water, (**b**) micro-nano bubble water.

The impact of SF on the dynamic elastic modulus of freeze-thaw C60 concrete is depicted in Figure 8, considering the use of tap water and micro-nano bubble water during mixing. The optimal frost resistance of C60 concrete can be achieved with a 7% SF content. Under the use of ordinary mixing water, the relative dynamic elastic modulus of the C60-G4 and C60-G7 groups are comparable at 7% SF content. The minimum relative dynamic elastic modulus of C60-G7 is 0.7924, exhibiting a 5.9% improvement over the blank group. However, the relative dynamic elastic modulus of C60-G10 is the lowest, even lower than that of the blank group at 0.7328. Incorporating WNBW as the mixing water, the use of C60-GW7 concrete yields a dynamic elastic modulus of 0.8158, while C60-G7 concrete produces a 0.7924 dynamic elastic modulus result. With WNBW as the mixing water, the loss of the dynamic elastic modulus of the concrete is reduced by 2.9%. The implementation of WNBW has been shown to impact the frost resistance of C60 concrete positively.

#### 3.6.3. Characteristic Parameters of Pore Structure

The assessment of concrete durability during freeze–thaw cycles relies on the identification of the characteristic parameters of concrete pores. In light of this, the figure presents analysis data on concrete pore typical parameters after 100 freeze–thaw cycles. This analysis involves the examination of pore chord length structure [41], which is categorised into micropores (chord length < 50 µm), small pores (50 µm < chord length < 100 µm), mesopores (100 µm < chord length < 500 µm), and macropores (chord length > 500 µm). Moreover, the present study investigates the potential impact of WNBW and SF on the pore characteristic parameters of concrete post-freeze–thaw. Among them, the larger the proportion of pores with a diameter less than 50 um, the better the frost resistance of concrete.

**Figure 9:**
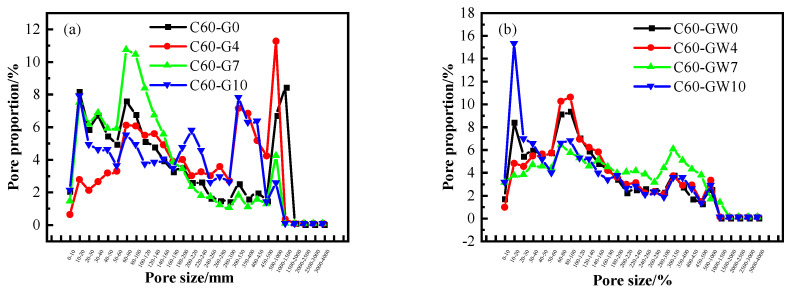
Pore structure characteristic parameters of concrete: (**a**) ordinary water, (**b**) micro-nano bubble water.

The Figure 9 presented exhibits the pore structure characteristic parameters of C60 concrete following 100 freeze–thaw cycles under varying SF content while using ordinary water as the mixing water. It can be inferred that under the 4% SF, the concrete possesses the fewest macropores. In descending order, the proportion of macropores was observed to be 15.17%, 11.52%, 4.2%, and 2.52% for the four SF contents, respectively. Moreover, the inclusion of WNBW as the mixing water resulted in a significant increase in the number of <500 um pore diameter chords, along with a significant decrease in the number of macropores. Furthermore, with the increase in WNBW, the proportion of macropores decreased to 2.49%, 3.25%, 3.85%, and 2.85%, respectively, which was noticeably lower than that of the C60 concrete mixed with ordinary water. Lastly, it was observed that the C60 concrete with 7% SF and 100% WNBW presented the least number and size of pores, hence providing the highest level of frost resistance after freeze–thaw [23].

#### 3.6.4. Pore Size Distribution

**Figure 10:**
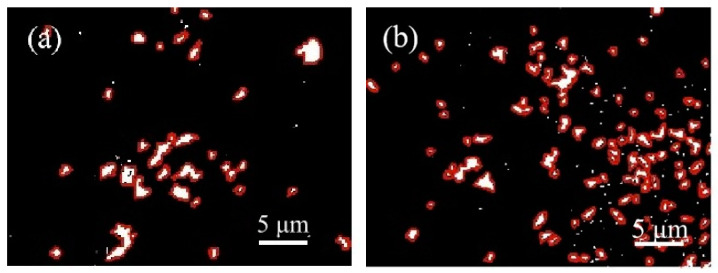
Pore distribution of tap-water-mixed concrete. (**a**) C60-G0, (**b**) C60-G7.

**Figure 11:**
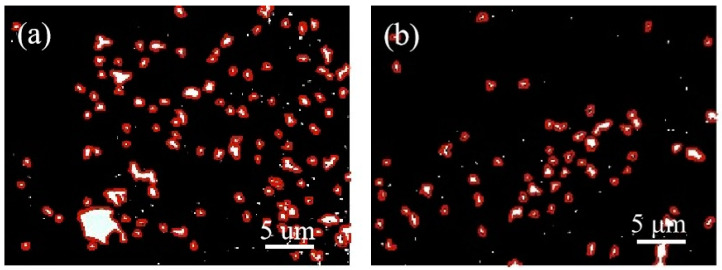
Pore distribution of WNBW mixed concrete. (**a**) C60-GW0, (**b**) C60-GW7.

By analysing the pore size distribution of the concrete following the freeze–thaw cycle, it is possible to assess the extent of internal structural damage. The Figure 10 and Figure 11 display the pore distribution map of C60 concrete prepared using ordinary water and WNBW after freeze–thaw, with varying SF content. To accentuate the contrast in the image, the chosen concrete for testing was C60, with varying SF content percentages of 0% and 7%. In this image, the black areas correspond to hardened concrete, while the white areas represent pores filled with nano-BaSO_4_. Therefore, the distribution of the white portions provides an accurate insight into the separation of pores in the concrete specimens following freeze–thaw cycles.

Upon examination of the figure, it is evident that the white area in the C60 concrete is reduced under varying mixing conditions, accompanied by a significant decrease in the number and size of the macropores. This can be attributed to the low water–cement ratio of C60 concrete, which leads to a densely packed internal structure, allowing minimal water penetration and mitigating freeze–thaw damage. Following 200 freeze–thaw cycles, C60 concrete exhibits lower internal porosity, more uniform pore distribution compared to the blank group (without SF), and better pore structure. Incorporating WNBW disperses the overall pore distribution in C60 concrete, characterised by fewer macropores, more micropores, and virtually no white flake pores. The dual use of WNBW and SF effectively sustained low porosity levels in the concrete. Combining SF and WNBW can be an effective approach to reduce the number and size of pores [42] in freeze–thaw concrete.

## 4. Conclusions

The primary focus of this study was to analyse the impact of WNBW and SF on the performance of C60 concrete. After thorough examination and investigation, the following conclusions can be drawn:

(1) The introduction of micro-nano bubble water and silica fume as additives in the C60 concrete mixture resulted in a reduction in slump and expansion. The observed decrease, although present, does not significantly impact the utility of fresh concrete. The results of the V-shaped funnel test revealed that micro-nano bubble water has a significant impact on the viscosity of C60 concrete, resulting in a 40% reduction. As per the aforementioned study, it can be inferred that the incorporation of micro-nano bubble water and silica fume in C60 concrete results in the reduced viscosity of high-strength concrete, thereby facilitating the transportation and construction of real-world projects. The reason for this phenomenon is attributed to the introduction of a significant quantity of micro-nano bubbles into the concrete mixture. This is beneficial, as these bubbles act as ball bearings, effectively reducing the inter-component friction within the concrete.
(2) Incorporating SF into concrete can reduce the cement content and subsequently lower the 3 d compressive strength of C60 concrete. SF functions as a filling agent within the concrete material during the early stages. The compressive strength of C60 concrete, as characterised by its 7-day and 28-day compressive strength, exhibited an initial increase followed by a decrease with the escalating content of SF. To achieve optimal results, a 7% SF volume fraction is recommended for C60 concrete. According to the findings, the compressive strength of concrete produced using WNBW exhibits superior performance compared to concrete produced with ordinary water over 3, 7, and 28 d. By using micro-nano bubble water as the mixing water for concrete, there is an enhancement in the powder’s dispersion. This results in an increased probability of the powder colliding with the mixing water, leading to a higher reaction speed and degree of hydration reaction.
(3) The electric flux test results revealed that the C60 concrete, blended with 100% WNBW and 7% silica fume, exhibits the lowest electric flux reading of 321.32 C, indicating optimal impermeability. The obtained mechanism aligns with that derived from the strength test. Concrete blended with micro-nano bubble water and silica fume exhibits a compact structure.
(4) Based on the results of the study, the incorporation of 100% WNBW into the concrete mix, which comprises 7% SF, has been determined to provide the most effective protection against carbonation for C60 concrete. It has been suggested that this success may be attributed to the favourable distribution of the pore structure within the resulting hardened concrete. The subsequent course of investigation entails a thorough examination and evaluation of the impact of WNBW and silica fume on the enduring carbonation performance of concrete.
(5) The frost resistance of concrete can be optimised using ordinary water with 7% SF content. The concrete with 4% and 7% SF exhibited a lower porosity and macropore content when compared to the blank group. However, a 10% SF content can decrease frost resistance in C60 concrete. On the other hand, when mixed with WNBW, the frost resistance of C60 concrete was observed to improve. The pore analysis diagram of the concrete reveals that the utilisation of micro-nano bubble water can effectively refine the pore structure of concrete, preserving a significant number of closed micro-pores and minimising the occurrence of macropores. This phenomenon serves to support the rationale behind the enhancement of concrete strength and durability, showcasing the potential benefits of incorporating such water into concrete production.
(6) The synergistic utilisation of WNBW and SF yields advantageous outcomes towards the transport and construction of C60 concrete, while simultaneously enhancing its compressive strength and durability. This paper offers theoretical substantiation for using the aforementioned approach in real-world engineering scenarios.

## Figures and Tables

**Table 1:** Chemical composition of cement, FA and SF (%).

Chemical Composition	Cement	FA	SF	GGBS
SiO_2_	21.14	53.97	89.24	36.23
Al_2_O_3_	5.38	31.15	2.30	14.64
Fe_2_O_3_	3.22	4.16	0.74	2.54
CaO	63.24	4.01	0.21	35.48
MgO	1.19	1.01	0.15	6.91
Na_2_O	0.28	0.89	1.03	0.06
K_2_O	0.54	2.04	1.07	0.97
SO_3_	2.34	0.73	1.90	2.15

**Table 2:** Physical parameters of sand.

Clay/%	Mud/%	Compacted Bulk Density/kg/m^3^	Loose Bulk Density /kg/m^3^	Apparent Density /kg/m^3^	Fineness Modulus
1.4	0.3	1680	1640	2760	2.6

**Table 3:** Physical parameters of stone.

Clay/%	Mud/%	Crush Index/%	Loose Bulk Density /kg/m^3^	Apparent Density /kg/m^3^	Needle-Like/%
0.3	0.1	7	1630	2620	6

**Table 4:** C60 concrete mix proportion with 35% FA (kg/m^3^).

Number	Cement	FA	GGBS	SF	River Sand	Stone	Water	WNBW
C60-G0	340	105	105	0	746	989	153	0
C60-G4	326	105	105	14	746	989	153	0
C60-G7	316	105	105	24	746	989	153	0
C60-G10	306	105	105	34	746	989	153	0
C60-GW0	340	105	105	0	746	989	0	153
C60-GW4	326	105	105	14	746	989	0	153
C60-GW7	316	105	105	24	746	989	0	153
C60-GW10	306	105	105	34	746	989	0	153

**Table 5:** Mixing concrete V-funnel through time.

Mixing Water Types	Tap Water				WNBW			
SF/%	0	4	7	10	0	4	7	10
V-funnel through time/s	107.5	114.7	121.4	146.2	64.5	69.9	69.3	72.4

**Table 6:** Influence of SF on the electric flux of C60 concrete.

SF/%	Tap Water/C	WNBW/C
0	711.12	459.03
4	363.56	321.32
7	403.08	405.68
10	422.64	489.72

## Data Availability

Data will be made available on request.
